# Supplementary material for: A single-cell atlas of the mouse and human prostate reveals heterogeneity and conservation of epithelial progenitors
Source: eLife. 2020 Sep 11;9:e59465. doi: 10.7554/eLife.59465 (PMC7529463; doi:10.7554/eLife.59465)
Supplement: Figure 4—source data 1. [file elife-59465-fig4-data1.docx]

**Figure 4—source data 1. Human prostate samples and corresponding clinical data**

| **Patient** | **Site** | **Age** | **Procedure** | **Overall diagnosis** | **Pathology of sample analyzed** | **Comments** |
| --- | --- | --- | --- | --- | --- | --- |
| 1 | Columbia | 70 | Cystoprostatectomy | High grade urothelial carcinoma of bladder | Benign prostatic hyperplasia with granulomatous prostatitis | scRNA-seq dataset #1 (mm037) |
| 2 | Columbia | 68 | Cystoprostatectomy | High grade urothelial carcinoma of bladder | Benign prostatic hyperplasia with chronic inflammation | scRNA-seq dataset #2 (mm033) |
| 3 | Columbia | 63 | Radical prostatectomy | Prostate adenocarcinoma (Gleason 3+3=6, pT2 N0) | Benign prostate with inflammation | scRNA-seq dataset #3 (mf002) |
| 4 | Cornell | 54 | Radical prostatectomy | Prostatic adenocarcinoma (Gleason 4+3=7, pT2 N0) | Benign prostate |  |
| 5 | Cornell | 65 | Radical prostatectomy | Prostatic adenocarcinoma (Gleason score 3+4=7, pT3a N1) | Benign prostate |  |
| 6 | Cornell | 79 | Cystoprostatectomy | High grade urothelial carcinoma of bladder | Benign prostate |  |
