# Supplementary material for: A single-cell atlas of the mouse and human prostate reveals heterogeneity and conservation of epithelial progenitors
Source: eLife. 2020 Sep 11;9:e59465. doi: 10.7554/eLife.59465 (PMC7529463; doi:10.7554/eLife.59465)
Supplement: Transparent reporting form [file elife-59465-transrepform.pdf]

Westbrook Centre, Milton Road
Cambridge CB4 1YG
UK
P 01223 855340
W elifesciences.org
T @elife

eLife's transparent reporting form

We encourage authors to provide detailed information *within their submission* to facilitate the interpretation and replication of experiments. Authors can upload supporting documentation to indicate the use of appropriate reporting guidelines for health-related research (see EQUATOR Network), life science research (see the BioSharing Information Resource), or the ARRIVE guidelines for reporting work involving animal research. Where applicable, authors should refer to any relevant reporting standards documents in this form.

If you have any questions, please consult our Journal Policies and/or contact us: editorial@elifesciences.org.

Sample-size estimation

- You should state whether an appropriate sample size was computed when the study was being designed
- You should state the statistical method of sample size computation and any required assumptions
- If no explicit power analysis was used, you should describe how you decided what sample (replicate) size (number) to use

Please outline where this information can be found within the submission (e.g., sections or figure legends), or explain why this information doesn't apply to your submission:

No statistical method was used to predetermine sample size. We followed the standard for single-cell studies of normal mouse and human tissues.

1. **Replicates**
   - You should report how often each experiment was performed
   - You should include a definition of biological versus technical replication
   - The data obtained should be provided and sufficient information should be provided to indicate the number of independent biological and/or technical replicates
   - If you encountered any outliers, you should describe how these were handled
   - Criteria for exclusion/inclusion of data should be clearly stated
   - High-throughput sequence data should be uploaded before submission, with a private link for reviewers provided (these are available from both GEO and ArrayExpress)

Please outline where this information can be found within the submission (e.g., sections or figure legends), or explain why this information doesn't apply to your submission:

eLife Sciences Publications, Ltd is a limited liability non-profit non-stock corporation incorporated in the State of Delaware, USA, with company number 5030732, and is registered in the UK with company number FC030576 andbranch number BR015634 at the address , UK | March 2019

- Multiple scRNA-seq datasets were generated for the mouse and human prostate (2 whole mouse prostate, 2 mouse anterior prostate, 2 mouse dorsal prostate, 2 mouse lateral prostate, 2 mouse ventral prostate, and 3 human benign prostate samples). Anatomical and histological studies were performed on at least 25 mice. Immunofluorescence microscopy studies were performed on at least 25 mice. Electron microscopy studies were performed on 2 mice. Flow cytometry studies were performed on at least 25 mice. Organoid studies were performed on at least 4 mice (ENR conditions) and 3-5 mice (HM conditions) per experiment for multiple experiments. Renal grafts used 3 to 5 mice per experiment with over 10 experiments performed. Histological and immunofluorescence microscopy studies were performed on 6 human benign prostate specimens.
- Single-cell sequencing data exclusion is extensively explained in the Methods section of our manuscript. It follows the standards of the field based on the cell quality.
- Single-cell RNA-sequencing data from this study have been deposited in the Gene Expression Omnibus (GEO) under the accession number GSE150692. Reviewers can access these data through the following private link with the token: kzexkkayhtsndar

  https://urldefense.proofpoint.com/v2/url?u=https-3A__www.ncbi.nlm.nih.gov_geo_query_acc.cgi-3Facc-3DGSE150692&d=DwIBAg&c=G2MiLlal7SXE3PeSnG8W6_JBU6FcdVjSsBSbw6gcR0U&r=fhFNgFUGMvi2oYHRn3inm3knY-9V2oygDjnaEr11xcU&m=nNjagcVpJ1pmI5rDKLcc_YsktGuD67DClQPwW0667mg&s=Eo9FtHtEr0gsNfYh9c1zrDVnzfEyv5hQ8LsD6rKsYw4&e=

Statistical reporting

- Statistical analysis methods should be described and justified
- Raw data should be presented in figures whenever informative to do so (typically when N per group is less than 10)
- For each experiment, you should identify the statistical tests used, exact values of N, definitions of center, methods of multiple test correction, and dispersion and precision measures (e.g., mean, median, SD, SEM, confidence intervals; and, for the major substantive results, a measure of effect size (e.g., Pearson's r, Cohen's d)
- Report exact p-values wherever possible alongside the summary statistics and 95% confidence intervals. These should be reported for all key questions and not only when the p-value is less than 0.05.

Please outline where this information can be found within the submission (e.g., sections or figure legends), or explain why this information doesn't apply to your submission:

Statistical information is provided within the figures and their corresponding legends. Methodology is detailed in the Methods section.

Group allocation

- Indicate how samples were allocated into experimental groups (in the case of clinical studies, please specify allocation to treatment method); if randomization was used, please also state if restricted randomization was applied
- Indicate if masking was used during group allocation, data collection and/or data analysis

Please outline where this information can be found within the submission (e.g., sections or figure legends), or explain why this information doesn't apply to your submission:

Only healthy mice or human benign prostate samples were considered in our analysis. Therefore, randomization and masking of research subjects was not required.

Additional data files ("source data")

- We encourage you to upload relevant additional data files, such as numerical data that are represented as a graph in a figure, or as a summary table
- Where provided, these should be in the most useful format, and they can be uploaded as "Source data" files linked to a main figure or table
- Include model definition files including the full list of parameters used
- Include code used for data analysis (e.g., R, MatLab)
- Avoid stating that data files are "available upon request"

Please indicate the figures or tables for which source data files have been provided:

- Source data is provided for figures 1 and 4.
- The following software has been used for data collection: Cell Ranger Single-Cell Software Suite (v.2.1.1, 10x Genomics), BD FACS Diva, NIS Elements (Nikon), LAS X Leica, Graphpad Prism.
- The following software has been used for data analysis:
  - FCS Express v. 7, ImageJ v. 1.52, Fiji v. 2.0.0, Graphpad Prism.
  - Python code sources:
  - Random Matrix Theory: https://rabadan.c2b2.columbia.edu/html/randomly/
  - Optimal Transport: https://github.com/rflamary/POT
  - Phylogenetic tree analysis: https://github.com/KlausVigo/phangorn

- Leiden algorithm for clustering (implemented in): https://scanpy.readthedocs.io/en/stable
